# Organspende und Organassessment nach primärem Herz-Kreislauf-Stillstand und sekundärem Hirntod

**DOI:** 10.1007/s00104-024-02094-x

**Published:** 2024-05-15

**Authors:** Philip C. Müller, Beat P. Müller, Philipp Dutkowski

**Affiliations:** 1 Klinik für Viszeralchirugie, Clarunis – Universitäres Bauchzentrum, Universitätsspital Basel, Basel, Schweiz; 2 Department of Surgery, Clarunis – University Digestive Health Care Centre Basel, Spitalstr. 21, 4031 Basel, Schweiz

**Keywords:** Organmangel, Lebertransplantation, Ethische Aspekte, Legale Aspekte, Hypotherme oxygenierte Maschinenperfusion, Organ shortage, Liver transplantation, Ethical aspects, Legal aspects, Hypothermic oxygenated machine perfusion

## Abstract

**Hintergrund:**

Der weltweite Organmangel ist das größte Hindernis, um die dringend notwendigen Lebertransplantationsaktivitäten auszuweiten. Um die Anzahl gespendeter Organe zu erhöhen, wurde in vielen europäischen Ländern neben der Spende nach Hirntod („donation after brain death“ [DBD]) auch die Spende bei primärem Herz-Kreislauf-Tod („donation after circulatory death“ [DCD]) und sekundärem Hirntod eingeführt.

**Ziel der Arbeit:**

Es erfolgt eine Zusammenfassung der legalen und ethischen Aspekte des Herz-Kreislauf-Todes, des Prozesses der DCD-Spende, der klinischen Ergebnisse insbesondere in Bezug auf das Organassessment vor einer geplanten DCD-Lebertransplantation.

**Ergebnisse:**

In Europa haben 11 Länder aktive DCD-Lebertransplantationsprogramme, und 2023 wurden in Europa insgesamt 1230 DCD-Lebertransplantationen durchgeführt. Den höchsten Anteil machten DCD-Lebertransplantationen in Belgien (52,8 %), Holland (42,8 %) und der Schweiz (32,1 %) aus. Die adäquate Selektion von Spendern und Empfängern ist bei DCD-Transplantationen entscheidend, und die Verwendung von DCD-Lebern hängt insbesondere von der Bereitschaft zur routinemäßigen Maschinenperfusion ab. Die Spitzenreiter Belgien, Frankreich und Italien implantieren rund 68–74 % aller DCD-Organe. Bei adäquatem Organassessment sind die Langzeitergebnisse von DBD- und DCD-Lebertransplantationen vergleichbar. Die hypotherme oxygenierte Maschinenperfusion (HOPE) erlaubt neben der Einschätzung des mitochondrialen Schadens gleichzeitig eine mitochondriale Protektion durch die Oxygenierung. Die Etablierung eines aeroben Stoffwechsels in Mitochondrien in der Hypothermie führt zu einer Reduktion toxischer Metabolite und zur Wiederherstellung der ATP(Adenosintriphosphat)-Speicher, dadurch kommt es anschließend bei der Implantation zu einer „Reperfusion Light“.

**Schlussfolgerungen:**

Die Erweiterung des Spenderpools durch DCD-Spender wirkt der weltweiten Organknappheit entgegen. Bei adäquater Selektion und routinemäßigem Organassessment sind sowohl Kurzzeit- als auch Langzeitergebnisse von DBD- und DCD-Lebertransplantationen vergleichbar.

## Abstract (en)

**Background:**

The global organ shortage is the biggest obstacle to expand urgently needed liver transplantation activities. In addition to donation after brain death (DBD), donation after primary circulatory death (DCD) has also been introduced in many European countries to increase the number of donated organs.

**Objective:**

This article summarizes the legal and ethical aspects of DCD, the practical donation process of DCD, the clinical results of DCD liver transplantation with a special focus on organ assessment before a planned DCD liver transplantation.

**Results:**

In Europe 11 countries have active DCD liver transplantation programs and a total of 1230 DCD liver transplantations were performed in Europe in 2023. The highest proportion of DCD liver transplantations were recorded in Belgium (52.8%), the Netherlands (42.8%) and Switzerland (32.1%). The adequate selection of donors and recipients is crucial in DCD transplantation and the use of DCD livers particularly depends on the preparedness of the healthcare system for routine machine perfusion. The leaders are Belgium, France and Italy which implant around 68–74% of DCD organs. With an adequate organ assessment, the long-term results of DBD and DCD liver transplantations are comparable. To assess mitochondrial damage and thus organ quality, hypothermic oxygenated machine perfusion (HOPE) was introduced and has the secondary benefit of mitochondrial protection through oxygenation. The establishment of aerobic metabolism in mitochondria under hypothermia leads to a reduction of toxic metabolites and the restoration of ATP storage, which subsequently leads to a reperfusion light during implantation.

**Conclusion:**

Expanding the donor pool with DCD donors can counteract the global organ shortage. With adequate patient selection and routine organ assessment short-term and also long-term outcomes of DBD and DCD liver transplantation are comparable.

Die Transplantation solider Organe ist seit der erstmaligen Nierentransplantation 1954 und der ersten Lebertransplantation 1963 eine medizinische Erfolgsgeschichte [1, 2]. Wichtige Bestandteile für die Transplantation der häufig sehr kranken Patienten sind neben einer adäquaten Immunsuppression eine zeitnahe Transplantation. Der weltweite Organmangel ist jedoch mit das größte Hindernis, um die dringend notwendigen Transplantationsaktivitäten auszuweiten. Im Bereich der Lebertransplantation ist die Wartezeit auf ein passendes Organ mit einer hohen Wartelistemortalität assoziiert.

Heutzutage werden in Europa die meisten Organe von Spendern nach Hirntod („donation after brain death“ [DBD]) entnommen [3]. Aufgrund des Organmangels wurden über die letzten Jahrzehnte unterschiedliche Strategien implementiert, um die Anzahl gespendeter Organe zu erhöhen. Einerseits wurden nationale Gesetze (z. B. Wiederspruchlösung) und Richtlinien angepasst, andererseits werden häufiger sog. Extended-criteria-Donoren (ECD) akzeptiert. ECD unterscheiden sich in Bezug auf Alter, Steatosegrad der Leber, vorhandene virale Infekte oder warme Ischämiezeit wie z. B. durch die Spende bei primärem Herz-Kreislauf-Tod („donation after cardiac death“ [DCD]). Gemäß neusten Ergebnissen könnte die Einführung der DCD-Spende in Deutschland selbst mit defensiven Berechnungen zu zusätzlichen 1500 Leberangeboten führen [4]. Trotz dieser potenziell deutlichen Erweiterung des Spenderpools werden DCD-Organe aufgrund potenziell eingeschränkter Organfunktion von Transplantationszentren häufig direkt und ohne weiteres Organassessment abgelehnt. Interessanterweise sind die Ablehnungsraten von DCD-Organen sehr variabel und in Amerika sowie England (70–80 %) besonders hoch. Demgegenüber stehen Länder wie Spanien, Italien, Frankreich oder die Schweiz DCD-Organen mit Ablehnungsraten von 30–40 % deutlich offener gegenüber [5]. Neben nationalen Richtlinien zur Akzeptierung von DCD-Lebern wird in den letztgenannten Ländern häufig routinemäßig ein Organassessment mittels Maschinenperfusion durchgeführt, was nachweislich zu einer verbesserten „utilization rate“ führt.

Dieser Übersichtsartikel beleuchtet neben den legalen und ethischen Aspekten des Herz-Kreislauf-Todes den Prozess der DCD-Spende, die klinischen Ergebnisse der DCD-Lebertransplantation und geht auf die Wichtigkeit des Organassessments vor einer geplanten DCD-Transplantation ein.

## Legale und ethische Aspekte

Eine postmortale Organspende kommt sowohl bei Personen nach „primärem Hirntod“ durch eine direkte Schädigung des Gehirns (DBD), aber auch nach „sekundärem Hirntod“ infolge eines irreversiblen Herz-Kreislauf-Stillstands (DCD) infrage. Die Irreversibilität des Herz-Kreislauf-Stillstands ist ein wichtiges Kriterium unseres Todesverständnisses und bildet die ethische Grundlage der sog. „Non heart-beating donor“-Organspende. Grundvoraussetzung für die Organspende nach primärem Herz-Kreislauf-Stillstand ist eine Therapiezieländerung von Kuration auf Palliation. Diese Entscheidung wird unabhängig und vor der Beurteilung der Möglichkeit einer Organspende getroffen. Gemäß modifizierter Maastricht-Klassifikation werden DCD-Spender zudem weiter in erwartete und unerwartete Organspender unterteilt. Bei plötzlichem Kreislaufstillstand in oder außerhalb des Krankenhauses tritt der Tod unerwartet ein. Beim Beenden von lebenserhaltenden Maßnahmen hingegen wird der Tod erwartet (Tab. 1).

Grundvoraussetzung ist eine Therapiezieländerung von Kuration auf Palliation

**Tab. 1:** Einteilung der DCD(„donation after circulatory death“)-Spende gemäß modifizierter Maastricht-Klassifikation [6]

Kategorie	Definition	Organentnahme
I	Tod aufgefunden	Unkontrolliert
II	Beobachteter Tod	Unkontrolliert
III	Tod nach Abbruch von lebenserhaltenden Maßnahmen	Kontrolliert
IV	Kreislaufstillstand bei vorgängigem Tod infolge primärer Hirnschädigung	Unkontrolliert-kontrolliert

In Europa gibt es in den meisten Ländern bindende Gesetzgebungen zur DCD-Spende, zusätzlich sind häufig nationale, rechtlich nicht bindende Richtlinien vorhanden [7]. Interessant ist die unterschiedliche Regelung der sog. „No-touch“-Dauer, der Zeit zwischen dem Herz-Kreislauf-Stillstand und Todesfeststellung. Diese beträgt in den meisten Ländern 5 min und betrug in der Schweiz bis 2017 10 min, wurde dann auf 5 min reduziert. Die längste „No-touch“-Zeit ist in Italien mit 20 min vorhanden und trägt relevant zur funktionellen warmen Ischämiezeit (fWIT) bei.

## Prozess der Herz-Kreislauf-Tod-Spende

**Abb. 1:**
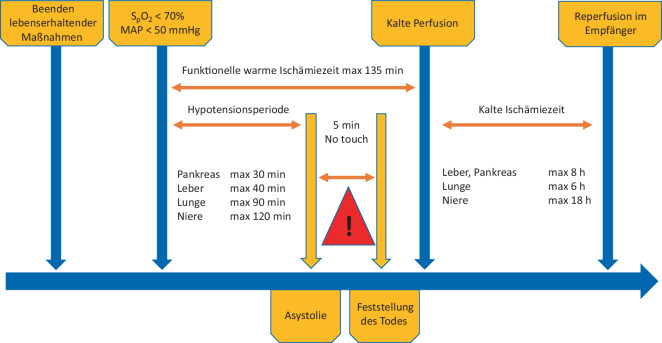
Schematische Darstellung des DCD(„donation after cardiac death“)-Spendeprozesses vom Beenden lebenserhaltender Maßnahmen bis hin zur Feststellung des Todes sowie der Reperfusion mit kalter Spüllösung im Empfänger. *MAP* mittlerer arterieller Blutdruck, *S*_*p*_*O*_*2*_ pulsoxymetrisch gemessene Sauerstoffsättigung

Nach Therapiezieländerung von Kuration auf Palliation erfolgt die Therapieumstellung je nach nationaler Richtlinie auf der Intensivstation oder im Operationssaal. Nach Einstellen der lebenserhaltenden Maßnahmen beginnt der Sterbeprozess mit Entsättigung und Herz-Kreislauf-Versagen und damit die fWIT. Obwohl die fWIT als Konzept international akzeptiert ist, gibt es keine einheitliche Definition davon, häufig wird der Start der fWIT ab einem mittleren Druck unter 50 mm Hg und einer Sättigung unter 70 % definiert. Der Prozess führt entweder zum Herz-Kreislauf-Stillstand oder aber der Patient verstirbt nicht im gesetzten Rahmen, sodass auch der Spendeprozess abgebrochen wird. Im Falle des Herz-Kreislauf-Stillstands folgt je nach nationalen Richtlinien nach dem echokardiographisch dokumentierten Herzstillstand eine „No-touch“-Phase und im Anschluss die Todesfeststellung gemäß nationaler Gesetzgebung [7]. Der Abschluss dieser Untersuchung entspricht dem Todeszeitpunkt. Zu diesem Zeitpunkt beginnt der Prozess der Organentnahme (Abb. 1).

Eine schnelle Organentnahme beeinflusst nachweislich die Organqualität, hier gilt es, neben einer zügigen Kanülation v. a. auf eine vollständige kalte Spülung der Leber zu achten und die makroskopische Erscheinung der Leber einzuschätzen. Bei der „Super-Rapid-Technik“ sollte die Aorta 2 min nach Laparotomie kanüliert werden und entsprechend mit der kalten Spülung der Organe begonnen werden. Im Anschluss sollte die Hepatektomiezeit so kurz wie möglich gehalten werden, da die Entnahmezeit eindeutig mit klinischen Ergebnissen korreliert. Eine Hepatektomie von > 60 min ist mit primärem Transplantatversagen und eingeschränktem Transplantatüberleben assoziiert [8]. Eine amerikanische Registerstudie mit insgesamt 3810 DCD-Hepatektomien zeigte, dass eine Hepatektomiedauer von > 42 min mit vermindertem Transplantat- und Patientenüberleben assoziiert war [9].

## DCD(„donation after circulatory death“)-Spende- und Transplantationsaktivität in Europa

In Europa haben 11 Länder aktive DCD-Lebertransplantationsprogramme, darunter Belgien, Tschechien, Frankreich, Italien, Holland, Spanien, Schweiz, Schweden, Norwegen, England und Österreich [3]. In anderen europäischen Ländern ist die Einführung von DCD-Programmen geplant (Dänemark, Kroatien) oder wird aufgrund fehlender Gesetzgebungen, Regulatorien bzw. fehlender Erfahrung nicht durchgeführt. Der Weg von einem DCD-Organangebot bis zur Implantation ist in den einzelnen Ländern sehr unterschiedlich geregelt, und den Gesundheitssystemen stehen ungleiche Ressourcen für diesen Prozess zur Verfügung. Entsprechend zeigen sich in der „utilization rate“, die das Verhältnis von angebotenen zu implantierten DCD-Lebern beschreibt, deutliche Unterschiede. Spitzenreiter in der Verwendung von DCD-Lebern sind Belgien, Frankreich und Italien mit einer „utilization rate“ von 68–74 % während in England (18,9 %) und Holland (28,4 %) nur ein Bruchteil der angebotenen Organe implantiert wird. Interessanterweise scheint die „utilization rate“ mit der Bereitschaft zur Maschinenperfusion zusammenzuhängen (s. Abschnitt „Organassessment vor DCD(„donation after cardiac death“)-Transplantation“). In Frankreich und Italien werden die DCD-Organe routinemäßig mit normothermer regionaler Perfusion (NRP) bzw. hypothermer oxygenierter Maschinenperfusion (HOPE) perfundiert. Demgegenüber wird die Maschinenperfusion in England (< 10 %) und Holland (20–30 %) bei einer Minderheit von DCD-Lebern eingesetzt [5].

Die „utilization rate“ scheint mit der Bereitschaft zur Maschinenperfusion zusammenzuhängen

Im Jahr 2023 wurden in Europa insgesamt 1230 DCD-Lebertransplantationen durchgeführt. Den höchsten Anteil machten DCD-Lebertransplantationen in Belgien (52,8 %), Holland (42,8 %) und der Schweiz (32,1 %) aus. Nachdem initial Sorgen um höhere Komplikationsraten bei DCD-Organen bestanden, zeigte sich interessanterweise bei den meisten europäischen Ländern eine Stagnation von DBD-Transplantationen bei gleichzeitiger Verdopplung der implantierten DCD-Lebertransplantationen allein im Zeitraum von 2018 bis 2023 (Tab. 2).

**Tab. 2:** Nationale Angaben zu DCD(„donation after cardiac death“)-Lebertransplantationen (LT) in Europa

Land	Nationale Gesetzgebung	DCD-Kategorie^a^	No-touch-Zeit	Ante-mortem-Kanülierung	Maschinenperfusion [5]	Spender (pmp)	LT (*n*)	DCD (*n*)	% DCD 2023	% DCD 2018	Zunahme 2018–2023
Belgien	Ja	Kontrolliert Unkontrolliert	5	Ja	Selektiv HOPE, NRP	35,8	314	166	52,8	25,3	+ 27,5
England	Ja	Kontrolliert	5	Nein	Selektiv NRP, NMP	21,1	911	252	27,6^a^	22,7	+ 4,9
Frankreich	Ja	Kontrolliert Unkontrolliert	5	Ja	Routine NRP	25,8	1371	161	11,7	3,5	+ 8,2
Holland	Nein	Kontrolliert Unkontrolliert	5	Nein	Selektiv HOPE, NRP, NMP	17,3	208	89	42,8	40,8	+ 2
Italien	Ja	Kontrolliert Unkontrolliert	20	Ja	Routine NRP + HOPE	25,5	1592	115	7,2	2,8	+ 4,4
Norwegen	Nein	Kontrolliert	5	Ja	Routine NRP	20,2	92^a^	4	4,3	0	+ 4,3
Österreich	Nein	Kontrolliert Unkontrolliert	10	Ja	–	21,4	117	6	5,1^a^	1,7	+ 3,4
Schweden	Nein	Kontrolliert	5	Nein	Routine NRP	20,9	198	10	5,1	0	+ 5,1
Schweiz	Nein	Kontrolliert Unkontrolliert	5^a^	Nein	Routine HOPE, NRP	22,7	137	44	32,1	14,4	+ 17,7
Spanien	Ja	Kontrolliert Unkontrolliert	5	Ja	Selektiv NRP	46,0	1191	371	31,2	15,9	+ 15,3
Tschechien	Ja	Kontrolliert Unkontrolliert	10	Nein	–	28,5	189	12	6,3	0	+ 6,3

*HOPE* hypotherme oxygenierte Maschinenperfusion, *NMP* normotherme Ex-situ-Perfusion, *NRP* normotherme regionale Perfusion

^a^Gemäß Maastricht-Klassifikation

## Spender-Empfänger-Matching bei DCD(„donation after circulatory death“)-Organen

Die adäquate Selektion von Spendern und Empfängern ist bei DCD-Transplantationen entscheidend. Da DCD-Organe bereits durch den Sterbeprozess bei primärem Herz-Kreislauf-Tod einen relevanten ischämischen Schaden erleiden, ist die Empfängerselektion umso wichtiger. Studien aus England und Amerika zeigen, dass unterschiedliche empfängerspezifische Faktoren mit einem verminderten Transplantatüberleben assoziiert sind: Intensivpflichtiger (IPS) oder beatmeter Empfänger, höherer „Model for end stage liver disease“(MELD)-Score, Alter bzw. Retransplantationen [10, 11]. Um die Ergebnisse von DCD-Lebertransplantationen zu optimieren, werden deshalb prinzipiell unterschiedliche Risikoanpassungen empfohlen:

1. Minimierung der chirurgischen Komplexität, indem zeitaufwendige Hepatektomien und komplexe vaskuläre Rekonstruktionen vermieden werden,
2. Auswahl von stabilen Spendern, die eine kurzfristig eingeschränkte Organfunktion bei marginalen DCD-Organen tolerieren. Hier sollte insbesondere auf einen fehlenden bzw. niedrigen Vasopressorenbedarf und auf eine fehlende künstliche Beatmung geachtet werden.

Um die genannten Risikofaktoren adäquat gegeneinander abzuwägen, haben sich diverse DCD-spezifische Risk-Scores etabliert, wie der UK DCD-Risk Score [10], der UCLA-DCD Score [12] und der DCD Risk Index [13]. Insbesondere der UK DCD-Risk Score führt anhand von 7 Parametern zu einer granularen Stratifizierung in 3 Gruppen mit unterschiedlichem Transplantatüberleben. Berücksichtigt werden 2 Spenderfaktoren (Alter und Body Mass Index [BMI]), 2 Entnahmefaktoren (fWIT und kalte Ischämiezeit) sowie 3 Empfängerfaktoren (Alter, MELD, Retransplantation). Dadurch kommt ein Score von 0 bis 27 Punkten zustande, wobei in der Low-risk-Gruppe (≤ 5 Punkte) das 1‑Jahres-Graft-Überleben bei > 95 %, bei moderatem Risiko (6 bis 10 Punkte) bei > 85 % und in der Futile-Gruppe (> 10 Punkte) bei < 40 % liegt [10].

Die adäquate Selektion von Spendern und Empfängern ist bei DCD-Transplantationen entscheidend

Das tolerierte Spender-Empfänger-Risiko ist international sehr variabel und v. a. auch von der erwarteten Wartezeit abhängig. Im Schnitt warten Empfänger in Spanien 1,5 Monate, während man in der Schweiz oder Italien 10 bzw. 18 Monate auf eine Leber wartet. Dementsprechend besteht in Spanien eine geringere Risikobereitschaft bei jedoch exzellenter „utilization rate“ von Niedrig- bis mittleren Risikoorganen. Demgegenüber besteht in Italien oder der Schweiz ein deutlich höherer Druck, auch Hochrisiko-DCD-Organe zu akzeptieren [5].

## Klinische Ergebnisse der DCD(„donation after circulatory death“)-Lebertransplantation

Gemäß den eben genannten spender- und empfängerspezifischen Risikofaktoren umfassen die klinischen Resultate von DCD-Lebertransplantationen eine heterogene Gruppe. Für die Interpretation klinischer Resultate gilt es zudem zu berücksichtigen, ob die Organe präoperativ mittels Maschinenperfusion beurteilt wurden oder nicht. Differenzierte Ergebnisse hierzu stammen aus einer bizentrischen Studie, die 3 Patientengruppen mit je 50 Patienten verglich:

1. DCD-Transplantation ohne Maschinenperfusion,
2. DCD mit HOPE und
3. DBD.

Von den spenderspezifischen Angaben war auffällig, dass die DCD + HOPE-Donoren signifikant älter waren, eine längere fWIT hatten sowie einen höheren Verfettungsgrad aufwiesen. Interessanterweise war die Rate an PNF („primary non function“) (DCD 4 % vs. DCD + HOPE 0 % vs. DBD 2 %), arteriellen Komplikationen (DCD 12 % vs. DCD + HOPE 8 % vs. DBD 6 %) und Galleleckagen (DCD 2 % vs. DCD + HOPE 2 % vs. DBD 4 %) zwischen den Gruppen nicht unterschiedlich. Allerdings zeigten sich in der Gruppe der DCD ohne HOPE eine erhöhte Rate an ischämischen Strikturen (DCD 22 % vs. DCD + HOPE 8 % vs. DBD 2 %) und ein erhöhtes Transplantatversagen (DCD 36 % vs. DCD + HOPE 14 % vs. DBD 6 %). Diese Unterschiede waren bei DCD-Organen mit HOPE im Vergleich zu DBD Transplantationen nicht vorhanden [14]. Die Langzeitergebnisse und Lebensqualität nach DCD- und DBD-Transplantation wurden in einer großen Propensity-Score-gematchten Analyse von jeweils 300 Patienten verglichen. Es zeigt sich weder beim 1‑, 3‑ oder 5‑Jahres-Transplantatüberleben ein Unterschied (DCD: 83,8 %, 75,5 % und 70,1 % vs. DBD: 88,4 %, 80,3 % und 73,9 %; *p* = 0,27) noch beim 1‑, 3‑ oder 5‑Jahres-Patientenüberleben (DCD: 92,3 %, 86,1 % und 80,3 % vs. DBD: 92,3 %, 85,1 % und 79,5 %; *p* = 0,81). Zusätzlich zeigte sich ebenfalls kein Unterschied in Bezug auf die physische und mentale Lebensqualität (physisch: 44 vs. 45 Punkte; *p* = 0,34 und mental: 52 vs. 52 Punkte; *p* = 0,83) [15].

In einer Benchmark-Studie mit 1012 Low-risk-DCD-Transplantationen wurden Referenzwerte von DCD-Lebertransplantationen anhand von 17 internationalen Zentren festgelegt. In das Low-risk-Kollektiv wurden Patienten ohne akutes Leberversagen, mit einem MELD-Score von ≤ 20 Punkten, die nicht beatmet oder an der Dialyse waren, sowie Organe mit einer fWIT von ≤ 30 min eingeschlossen. In dieser selektionierten Patientengruppe konnten exzellente postoperative Ergebnisse gezeigt werden mit Referenzwerten für PNF ≤ 2,5 %, Galleleckage ≤ 8,3 %, postoperative Dialyse ≤ 9,6 % und Spitalaufenthalt ≤ 16 Tage. Die gefürchteten biliären Langzeitprobleme ischämische Cholangiopathie (Benchmark cut-off ≤ 16,8 %) und Anastomosenstrikturen (Benchmark cut-off ≤ 28,4 %) waren eindeutig von der warmen Ischämie der Organe abhängig. Bei einer fWIT > 30 min lagen die ischämische Cholangiopathierate mit 21 % (Benchmark cut-off ≤ 16,8 %) und auch das 1‑Jahres-Transplantatversagen mit 23,5 % (Benchmark cut-off ≤ 14,4 %) deutlich über den DCD-Referenzwerten. Im 1‑Jahres-Follow-up wurden in der Benchmark-Kohorte vergleichbare Langzeitergebnisse zu DBD-Organen beschrieben. So waren insbesondere die Referenzwerte für Transplantatversagen ≤ 14,4 % (DBD-Benchmark ≤ 11 %) und Mortalität ≤ 9,6 % (DBD-Benchmark ≤ 9 %) vergleichbar, während jedoch eine erhöhte Retransplantationsrate von ≤ 6,9 % (DBD-Benchmark ≤ 4 %) für DCD-Organe beschrieben wird [16, 17].

## Organassessment vor DCD(„donation after circulatory death“)-Transplantation

Ein Schlüsselpunkt in der Verwendung von DCD-Organen ist eine objektive und verlässliche Beurteilung der Organqualität. Selbst 60 Jahre nach der ersten Lebertransplantation beruht die Beurteilung neben Laborparametern und Histologie des Spenders meist auf dem Bauchgefühl des Transplantationschirurgen. Durch die Einführung der Maschinenperfusion eröffnet sich jedoch die ideale Möglichkeit der objektiveren Organbeurteilung vor einer Transplantation. Anhand einer internationalen Multicenterkohorte von 34.269 angebotenen DCD-Lebern wurde gezeigt, dass die Maschinenperfusion einen hochrelevanten Einfluss auf die „utilization rate“ der angebotenen Organe hat. Die „utilization rate“ war in Ländern mit routinemäßiger Maschinenperfusion doppelt so hoch im Vergleich zu Ländern mit selektiver Perfusion (64,9 % vs. 28,4 %) [5]!

**Abb. 2:**
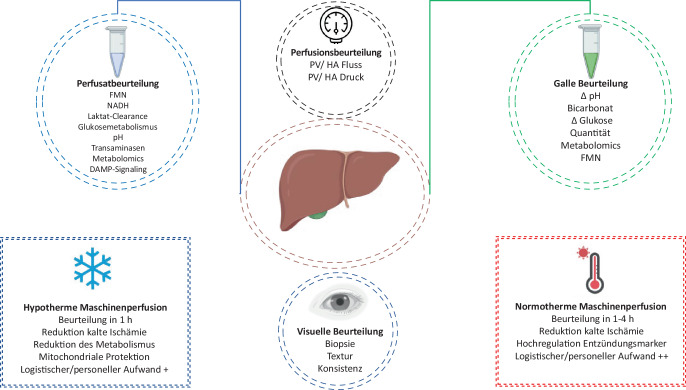
Organbeurteilung während der hypo- und normothermen Maschinenperfusion. *FMN* Flavin-Mononukleotid, *NADH* Nicotinamidadenindinukleotid, *DAMP* „damage-associated molecular pattern“, *PV* „portal vein“, *HA* „hepatic artery“

Ursächlich hierfür ist die Möglichkeit der Organbeurteilung während der Maschinenperfusion. So können die metabolische Funktion sowie der entstandene zelluläre Schaden durch den Spendeprozess beurteilt werden (Abb. 2). Klassischerweise zählen hierzu der zeitliche Verlauf der Parameter Laktat, Transaminasen, pH, Qualität und Quantität der Galleproduktion. Neben den klassischen Parametern können jedoch auch Moleküle des mitochondrialen Stoffwechsels wie mitochondriales Flavin, sog. Flavin-Mononukleotid (FMN), Nicotinamid-Adenin-Dinukleotid (NAD), Inosinmonophosphat (IMP) und Hypoxanthin gemessen werden. FMN wird dabei bei mitochondrialem Komplex-I-Schaden ausgeschüttet und kann in Echtzeit mittels Fluoreszenzspektroskopie gemessen werden. Dadurch ist eine Einschätzung des mitochondrialen Schadens und damit auch der Organfunktion während der Maschinenperfusion möglich.

In einer detaillierten Analyse von 54 Lebertransplantationen mit Organassessment mit HOPE konnte eine starke Korrelation von FMN mit postoperativen Koagulationsfaktoren (INR [International Normalized Ratio]: R = 0,75) und Peak Transaminasen (Peak ALT [Alanin-Aminotransferase]: R = 0,64) sowie zur Vorhersage von Early-allograft-Dysfunktion (C-Statistik: 0,72) gezeigt werden. Zusätzlich korrelierten die FMN-Werte mit klinisch relevanten Endpunkten wie Komplikationen (CCI [„comprehensive complication index“]: R = 0,68) und 3‑Monats-Transplantatverlust (AUC [„area under the curve“]: 0,93) [18].

Die Maschinenperfusion ermöglicht eine objektivere Organbeurteilung vor einer Transplantation

Es kommen grundsätzlich 3 unterschiedliche Techniken der Perfusion zur klinischen Anwendung:

1. hypotherme Maschinenperfusion,
2. normotherme Ex-situ-Perfusion und
3. normotherme regionale Perfusion.

### Hypotherme oxygenierte Maschinenperfusion

**Abb. 3:**
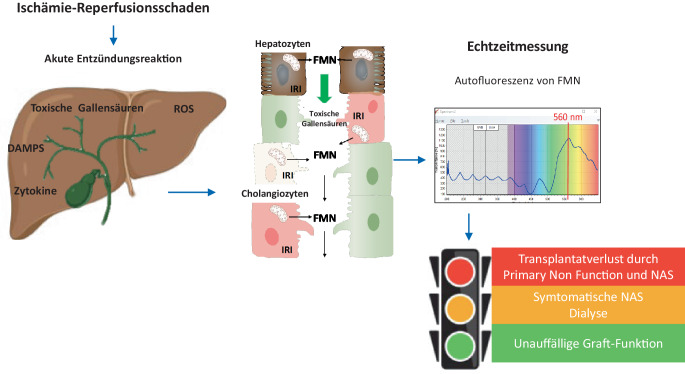
Während der hypothermen oxygenierten Maschinenperfusion (HOPE) werden der mitochondriale Schaden und die mitochondriale Funktion gemessen. Während der oxygenierten Reperfusion wird Flavin-Mononukleotid (FMN) von Komplex 1 ausgeschüttet und kann mittels Fluoreszenz gemessen werden. Die relativen FMN-Werte führen kontinuierlich zu einem erhöhten Komplikationsrisiko. *DAMPS* „damage-associated molecular patterns“, *ROS* Sauerstoffradikale, *NAS* „non anastomotic biliary strictures“

Bei der hypothermen Maschinenperfusion wird das Organ über die Pfortader (HOPE) oder über die Pfortader und die Leberarterie (Dual-HOPE) perfundiert und mit O_2_ (Sauerstoff) oxygeniert. Die Technik ist seit den 1970er-Jahren bekannt, gewann jedoch durch das Wissen um die mitochondriale Protektion durch O_2_ in den letzten Jahren international an Bedeutung [19]. Während des Sterbe- und Spendeprozesses kommt es zu einer Ischämie mit anaerobem Metabolismus und Akkumulation toxischer Metabolite. Wenn nach dieser Phase erneut Sauerstoff in den Metabolismus kommt, hat dies schwerwiegende Nebeneffekte, insbesondere wenn der Prozess unter normothermen Bedingungen erfolgt. Es kommt zur Freisetzung von inflammatorischen Zytokinen, „damage-associated molecular patterns“ (DAMPs) und Sauerstoffradikalen (ROS), dem wichtigsten Molekül der Ischämie-Reperfusion-assoziierten Entzündung (Abb. 3). Die Zugabe von Sauerstoff unter hypothermen Bedingungen führt jedoch über unterschiedliche protektive Signalwege zu einer „Reperfusion Light“. Insbesondere die Etablierung des aeroben Stoffwechsels in Mitochondrien in der Hypothermie führt zu einer Reduktion toxischer Metabolite (Nicotinamidadenindinukleotid [NADH], Succinat) und zur Wiederherstellung der ATP(Adenosintriphosphat)-Speicher [20].

Seit 2012 werden bei uns DCD-Organe routinemäßig vor der Implantation mittels HOPE beurteilt [21]. Die HOPE erfolgt endischämisch, d. h. nach Super-Rapid-Entnahme und Lagerung in gekühlter Spüllösung. Die Perfusion erfolgte über die Pfortader mit einem maximalen Pfortaderdruck von 3 mm Hg mit entsprechend niedrigem Pfortaderfluss von 150–300 ml/min. Als Perfusat wurden 3 l von hoch oxygenierter (80–100 kPa) University of Wisconsin-Perfusionslösung verwendet, und die HOPE wird mindestens für 1 h durchgeführt. Für das Organassessment während der HOPE wird das Perfusat analysiert. Wie bereits beschrieben, basieren unsere Kriterien zur Organannahme zu einem großen Teil auf den gemessenen FMN-Werten.

### Normotherme Ex-situ-Perfusion

Normotherme Ex-situ-Perfusion (NMP) erfolgt nach der Organentnahme in möglichst physiologischen Bedingungen mit 37° oxygeniertem, blutähnlichem Perfusat. Die NMP kann entweder direkt im Spenderspital durchgeführt werden, was den Transport von Maschinen und Material erfordert, jedoch die kalte Ischämie reduziert und zu vermindertem Organschaden führt [22]. Andererseits kann NMP auch endischämisch nach Organentnahme und Cold Storage durchgeführt werden. Die zweite Variante ist logistisch einfacher und günstiger, scheint jedoch mit weniger erfolgreicher Eindämmung des Ischämie-Reperfusionsschadens vergesellschaftet zu sein [23].

Während der NMP können sowohl Organfunktion als auch der Schaden evaluiert werden, was bei der Entscheidung der Organverwendung hilft. Die Organbeurteilung basiert hauptsächlich auf hepatozellulären oder biliären Kriterien [24]. Die Leberfunktion kann bezüglich metabolischer Funktion (Laktat, pH-Entwicklung) und exkretorischer Funktion (Galleproduktion, Gallezusammensetzung, Bikarbonat, Galle pH) analysiert werden. Zusätzlich kann die Hämodynamik während der Perfusion (Fluss, Druck) beurteilt werden. Am häufigsten wird der Parameter Laktat zur Organbeurteilung hinzugezogen, und typischerweise sind während der endischämischen NMP 3 Laktatphasen zu beobachten:

1. ein initialer Laktatanstieg in der ersten Stunde,
2. ein zügiger Rückgang innerhalb von 2 h,
3. Steady-State mit niedrigem Laktat für den Rest der Perfusion.

Obwohl diverse Studien einen Zusammenhang zwischen dem Laktatmetabolismus zeigen konnten, muss festgehalten werden, dass die meisten Lebern während der Maschinenperfusion Laktat abbauen und dieser Vorgang nicht vor eingeschränkter Organfunktion oder PNF schützt [25].

### Normotherme regionale Perfusion

NRP ist die Standardperfusionstechnik in Frankreich, Italien, und Spanien. Die NRP wird direkt nach dem Spendeprozess und der damit verbundenen warmen Ischämie durchgeführt. Falls thorakale und abdominale Organe entnommen werden, werden für die venoarterielle ECMO (extrakorporale Membranoxygenierung) die Aorta und der rechte Vorhof kanüliert. Falls nur abdominale Organe entnommen werden, werden A. und V. femoralis kanüliert. Im Anschluss wird das Abdomen mit oxygeniertem Spenderblut unter normothermen Konditionen für 2–4 h perfundiert. Während der NRP können analog zur NMP unterschiedliche hepatozelluläre Marker (Laktat, Transaminasen, pH) zur Evaluation der Organschädigung gemessen werden. Falls die Lebern die Viability-Kriterien erfüllen, erfolgt die Standardorganentnahme nach kalter Organperfusion. Anschließend erfolgt der Transport zum Empfängerzentrum in kalter Preservationslösung. Der Vorteil der NRP liegt in einer frühen Reperfusion und Reoxygenation bei Absenz von kalter Ischämie bei der Evaluation im Empfänger, zusätzlich können mehrere Organe mit derselben Technik perfundiert werden.

## Fazit für die Praxis

- Die Organspende nach primärem Herz-Kreislauf-Stillstand ist in den meisten europäischen Ländern etabliert und trägt in relevantem Maß zum Spenderpool bei.
- Die Organe nach primärem Herz-Kreislauf-Stillstand weisen durch den Spendeprozess und der damit einhergehenden funktionellen warmen Ischämie einen Organschaden auf und gehören zu sog. Extended-criteria-Spendern.
- Nach primärem Herz-Kreislauf-Tod bestehen international eindrückliche Unterschiede zwischen angebotenen und implantierten Lebern („utilization rate“).
- Das Organassessment mittels Maschinenperfusion erhöht die „utilization rate“ nachweislich.
- Die hypotherme oxygenierte Maschinenperfusion erlaubt neben der Einschätzung des mitochondrialen Schadens gleichzeitig eine mitochondriale Protektion.
- Die Etablierung des aeroben Stoffwechsels in Mitochondrien in der Hypothermie führt zu einer Reduktion toxischer Metabolite und zur Wiederherstellung der ATP(Adenosintriphosphat)-Speicher, dadurch kommt es bei der anschließenden Implantation zu einer „Reperfusion Light“.
